# Gallbladder schistosomiasis as an uncommon cause of recurrent cholecystitis

**DOI:** 10.1016/j.idcr.2025.e02331

**Published:** 2025-07-27

**Authors:** Seid Getahun Abdela, Suleiman Ayalew Belay, Abdu Assen Ahmed

**Affiliations:** a Department of Internal Medicine, College of Medicine and Health Sciences, Wollo University, Dessie, Ethiopia; b School of Medicine, College of Medicine and Health Sciences, University of Gondar, Gondar, Ethiopia; c Department of Pathology, College of Medicine and Health Sciences, Wollo University, Dessie, Ethiopia

**Keywords:** Gallbladder schistosomiasis, Neglected tropical disease, *Schistosoma mansoni*, Recurrent cholecystitis

## Abstract

Gallbladder schistosomiasis is an extremely rare manifestation of *Schistosoma mansoni* infection, typically overshadowed by hepatic, intestinal, or urogenital involvement. We report a case of a 28-year-old woman from Ethiopia with a two-year history of recurrent right upper quadrant abdominal pain. Ultrasound suggested acalculous cholecystitis with a possible gallbladder polyp, and she underwent elective open cholecystectomy. Histopathology confirmed the presence of granulomatous inflammation and calcified *Schistosoma mansoni* ova embedded in the gallbladder wall. The patient recovered fully following praziquantel therapy.

## Case description

A 28-year-old woman from central Ethiopia presented with a two-year history of intermittent right upper quadrant (RUQ) pain associated with nausea and vomiting, particularly after fatty meals. She had been hospitalized multiple times for suspected acute cholecystitis, managed conservatively with intravenous fluids and antibiotics. Notably, she denied hematuria or gastrointestinal bleeding.

Physical examination revealed RUQ tenderness and a positive Murphy’s sign. Laboratory findings, including liver enzymes and viral hepatitis serologies, were within normal limits. Stool microscopy was negative for ova or parasites. Abdominal ultrasound revealed a thickened gallbladder wall and an echogenic intraluminal lesion suggestive of a polyp, but no gallstones—findings consistent with acalculous cholecystitis.

**Fig. 1:**
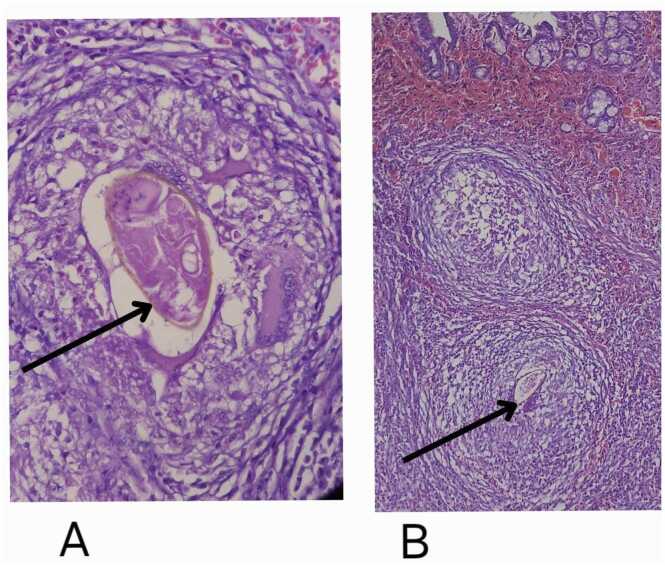
**A:** High-power (40 ×) microscopic image showing an epithelioid granuloma surrounding *Schistosoma* ova (shown in black arrow). **B:** Gallbladder mucosa with columnar epithelium and dense mononuclear inflammation, featuring multiple granulomas, one with a central *Schistosoma* egg (shown in black arrow).

An elective open cholecystectomy was performed. Gross inspection of the gallbladder revealed focal thickening of the mucosa. Histopathology revealed multiple well-formed granulomas containing calcified *Schistosoma mansoni* ova within the lamina propria and muscularis propria (Figs. 1A and 1B). No evidence of dysplasia or malignancy was found.

The patient received a single oral dose of praziquantel (40 mg/kg). She remained asymptomatic at six-month follow-up with no recurrence.

Gallbladder schistosomiasis is an uncommon and often underrecognized complication of *Schistosoma mansoni* infection, with few cases reported in the literature [1]. Unlike the frequently affected liver and genitourinary systems, gallbladder involvement typically presents with nonspecific symptoms that mimic common biliary diseases such as cholelithiasis or cholecystitis, leading to diagnostic challenges [2]. Ultrasound may reveal gallbladder wall thickening or polypoid lesions [3], but these findings are not definitive. Confirmatory diagnosis relies on histopathological identification of schistosome ova within the gallbladder wall. In endemic areas, clinicians should consider parasitic causes in patients with persistent or recurrent cholecystitis despite absence of gallstones. Cholecystectomy provides both diagnostic confirmation and symptom relief [4], while postoperative praziquantel therapy is crucial to eradicate residual infection and prevent recurrence.

## Statements

Informed consent was obtained from the patient.

## Authors’ statement

We confirm that the manuscript has been read and approved by all named authors and that there are no other persons who satisfied the criteria for authorship.

## CRediT authorship contribution statement

**Suleiman Ayalew Belay:** Writing – original draft, Data curation, Conceptualization. **Abdu Assen Ahmed:** Resources, Data curation, Conceptualization. **Seid Getahun Abdela:** Writing – original draft, Resources, Data curation, Conceptualization.

## Ethical approval

None.

## Consent

None.

## Funding

No funding was received.

## Declaration of Competing Interest

The authors declare that they have no competing interests in relation to this manuscript.

No financial, institutional, or personal conflicts of interest influenced the preparation or submission of this work.

Additionally, no external funding was received to support this study.
